# The association of prenatal adiposity characteristics with early childhood overweight and obesity: findings from a large and diverse mother–child cohort

**DOI:** 10.1038/s41366-026-02082-7

**Published:** 2026-04-14

**Authors:** Hua Min, Michael S. Bloom, Grace N. Lawrence, Alma Fuller, Kathi C. Huddleston

**Affiliations:** 1 Department of Health Administration and Policy, College of Public Health, George Mason University, Fairfax, VA USA; 2 Department of Global and Community Health, College of Public Health, George Mason University, Fairfax, VA USA; 3 School of Nursing, College of Public Health, George Mason University, Fairfax, VA USA

**Keywords:** Paediatrics, Endocrine system and metabolic diseases

## Abstract

**Background:**

Maternal prepregnancy body mass index (ppBMI) and gestational weight gain (GWG) are risk factors for overweight and obesity (OWO) in childhood. However, a better understanding of the magnitude, timing, and mediating mechanisms of these associations can inform interventions to improve maternal and child health.

**Methods:**

We conducted a prospective cohort study of 2899 mother–child dyads in northern Virginia (2012–2019). Maternal ppBMI was self-reported and GWG was calculated and categorized as excess (EGWG) using 2009 Institute of Medicine guidelines. Child weight was reported by parents every six months from birth to 24 months, and annually thereafter. Childhood OWO was defined as >85th percentile of WHO growth charts at 36 months. Generalized linear and mixed models estimated maternal OWO status and GWG as predictors of children’s body weight Z-score and growth velocity, adjusted for covariate. Causal mediation analysis was used to quantify birth weight and early growth velocity as intervening factors.

**Results:**

Mothers self-reported Hispanic (32.08%) and non-Hispanic (51.98%) ethnicity (15.94% missing). Mean ± SD ppBMI was 25.5 ± 5.4 kg/m² (35.46% with obesity) and GWG was 14.2 ± 6.8 kg (40.8% EGWG). At 36 months, 25.4% of children had overweight and 10.5% had obesity. Higher ppBMI (RR = 1.04 per 1.00 kg/m^2^; 95% CI: 1.03–1.06) and GWG was associated with childhood risk of OWO among non-Hispanic but not Hispanic mothers (interaction *P* = 0.02). Similarly, maternal obesity status (RR = 1.64; 95% CI: 1.26–2.13) and EGWG (RR = 1.38; 95% CI: 1.09–1.74) were associated with childhood OWO risk. Approximately 26.8% (95% CI: 14.9%–55.9%) of the ppBMI–OWO association could be attributed to birth weight.

**Conclusions:**

Maternal ppBMI and GWG were independently associated with risk of OWO in early childhood OWO, with variations by child age and ethnicity, highlighting the potential of pre- and perinatal interventions to reduce childhood obesity risk.

## Introduction

Childhood obesity, defined as a body mass index (BMI) ≥95th percentile for sex and age [1], has more than tripled in the US over the past 20 years [2]. In 2024, the US Centers for Disease Control (CDC) estimated that 14.7 million children aged 2–19 years had obesity. Obesity in childhood is particularly concerning because it tends to persist into adulthood. Nearly 90% of children with obesity at 3 years of age will have overweight or obesity in early adulthood [3, 4]. Furthermore, children with obesity experience increased risks of cardiovascular disease, dyslipidemia, insulin resistance, type 2 diabetes, and mental health issues, many of which persist into adulthood and contribute to greater long-term morbidity and mortality [4]. Identifying modifiable risk factors to prevent early obesity is therefore critical. Though inherited factors are important predictors of childhood obesity [5], the origins are complex and likely involve the interaction of genetic, behavioral, and environmental factors [6]. Among these factors, maternal health plays a major role.

Approximately 41.4% of U.S. women live with obesity. The prevalence of overweight (BMI >85th %ile for sex and age [1]) and obesity (OWO) varies by race and ethnic identity [7], and is disproportionately greater among Hispanic than non-Hispanic white women [8]. However, national obesity trends typically exclude pregnant women [9]. The high prevalence of obesity among women translates into increasing numbers of pregnant women with OWO, whose children may also be affected by obesity-related health risks. Maternal adiposity during pregnancy may influence fetal epigenetic patterning, increasing the risk of OWO in offspring due to the intergenerational transmission of obesity [3, 5, 10–12]. Maternal obesity influences infant birth weight, growth trajectory and rapid weight gain (RWG), which have been identified as risk factors for childhood obesity [13, 14].

The fetal environment and first 1000 days of postnatal life have been identified as a window of susceptibility to obesogenic risk factors [15, 16]. However, the relative importance of maternal obesity and gestational weight gain on childhood risk of OWO remain unclear, as do potential differences among different racial and ethnic groups. To help address the data gap, this study examines prepregnancy BMI (ppBMI), gestational weight gain (GWG), and self-reported ethnicity as predictors of early childhood OWO in an ethnically-diverse longitudinal birth cohort of 2899 mother–child dyads.

## Methods

### Study population

We enrolled pregnant people receiving prenatal care in a northern Virginia medical system between June 2012 and October 2019 as previously described in detail [17]. Parents were enrolled in this longitudinal study during pregnancy, followed throughout the prenatal and delivery period, and subsequently sent surveys to collect patient-reported outcome data as part of the First Thousand Days of Life Study, a trio-based longitudinal study examining genomic and environmental influences on child health that has been reported elsewhere. Briefly, inclusion criteria were women in their second trimester of pregnancy who were at least 18 years old, in generally good health, fluent and literate in English or Spanish, and willing to complete longitudinal follow-up surveys. Participants were enrolled during the routine second-trimester prenatal care visit and participants completed a baseline study questionnaire at enrollment, including self-reported prepregnancy maternal weight and height, as well as sociodemographic factors. Follow-up surveys collected information on maternal and child health, as well as mother-reported child weight and height, every six months for 24 months postpartum and annually thereafter. Mothers completed written informed consent and the study protocol was approved by the WCG Institutional Review Board (WCG protocol #20120204). To ensure reliable modeling of early childhood growth trajectories, this analysis included only mother–child dyads with a singleton term birth (≥37 weeks).

### Statistical analysis

We characterized the distributions of sociodemographic and clinical factors among mother–child dyads. We defined maternal ppBMI as self-reported prepregnancy body weight divided by self-reported height squared (kg/m^2^) and prepregnancy obesity as BMI ≥ 30 kg/m^2^. Maternal GWG was the difference in self-reported prepregnancy body weight and weight measured at the time of delivery in kilograms. We further categorized GWG as excessive gestational weight gain (EGWG) based on the 2009 Institute for Medicine guidelines [18].

We defined child body weight Z-score as parental reported body weight relative to the World Health Organization (WHO) Child Growth Charts for sex and age, using the “Healthy Birth, Growth & Development (HBGD)” package in the R statistical programming environment [19], and childhood OWO as weight >85 percentile of the sex-specific WHO Child Growth Chart for 36 months of age. In this study, we used weight-for-age rather than BMI-for-age or weight-for-height because there were a large number of missing height values. We defined growth velocity as the difference in body weight Z-score reported at 2 time points [17], and RWG as growth velocity Z-score >0.67 [20].

#### Multivariable models

We used generalized linear and mixed regression models to test the associations between ppBMI and GWG as continuous predictors of childhood OWO, child weight Z-score, child growth velocity, and RWG as study outcomes. In a second set of models, we used maternal prepregnancy obesity status and EGWG as categorical predictors of the study outcomes. We employed log-binomial regression to generate relative risks (RR) as effect estimates for OWO and RWG as dichotomous outcomes. However, we used a logistic regression model to generate odds ratios (OR) as effect estimates for dichotomous outcomes if the log-binomial model did not converge. We used linear regression for body weight Z-score and growth velocity as continuous outcomes. We included a random intercept on child to accommodate multiple correlated outcomes for growth velocity and RWG outcomes with repeated measures collected over 36 postnatal months. We adjusted the regression models for factors shown to predict ppBMI/GWG and childhood OWO/growth velocity reported by previous studies [13, 21–23], including maternal age at study enrollment (years), maternal education (no college, at least some college, graduate/professional school) as an indicator of socioeconomic position (SEP), self-reported maternal ethnic identity (non-Hispanic, Hispanic), infant sex assigned at birth (male, female), and gestational age at delivery (weeks).

We also conducted sensitivity analyses to evaluate the robustness of our results and interaction analyses to identify susceptible subgroups. We adjusted for year of birth as an additional covariate in a sensitivity analysis of time-trends and stratified by parity in a sensitivity analysis of siblingships. We tested interactions of ppBMI and GWG using likelihood ratios, in models that included cross-product terms with time of follow-up to identify heterogeneity in the associations with OWO, body weight Z-score, and growth velocity, and between maternal ethnicity, infant sex, low birth weight (<2500 g), or gestational age at delivery.

#### Mediation analysis

We implemented model-based causal mediation analysis to quantify birth weight and growth velocity from 0 to 6 months as mediators of associations between months ppBMI and GWG and OWO at 36 months [24]. First, birth weight or growth velocity were modeled as functions of ppBMI, GWG and other covariates in linear regression models. Next, OWO at 36 months was modeled as a function of birth weight or growth velocity, and ppBMI, GWG and other covariates in log-binomial regression models. Finally, we calculated the products of the regression coefficients from the separate regression models to estimate: 1. the indirect effects, or difference in OWO risk mediated by birth weight or growth velocity; 2. the direct effect, or difference in OWO risk not mediated by birth weight or growth velocity; and 3. the total effect, or overall difference in OWO risk. There was no interaction between ppBMI or GWG as predictors and birth weight or growth velocity as mediators, so we did not retain an interaction term in the model. We further estimated the indirect effect as the proportion of the total difference in OWO attributed to birth weight and growth velocity, adjusted for covariates and expressed as a percentile. We used the R package “mediate” [25] and 1000 bootstrap samples to provide 95% confidence intervals around the effect estimates.

For all multivariable analyses, we used the “Multiple Imputation by Chained Equations (mice)” package in the R statistical programming environment [26], with 10 imputed data sets, to account for attrition, cumulatively including *n* = 602 at 6 months, *n* = 916 at 12 months, *n* = 1144 at 18 months, *n* = 1418 at 24 months, and *n* = 1950 at 36 months of follow-up. We included all exposures, outcomes, and covariates as predictors of missing data (see Table 1). We also repeated multivariable regression models using a “complete case” approach as part of a sensitivity analysis, after excluding mother–child dyads with missing covariates, and compared the results to the imputed results. Finally, we used inverse probability of selection weights and generalized estimating equations (GEE) in a sensitivity analysis to assess the impact of loss to follow-up on the complete case results [27]. We defined statistical significance as *P*-value < 0.05 for main effects and *p*-value *P* < 0.10 for interactions. The R v.4.1.3 (R Foundation for Statistical Computing, Vienna, Austria) statistical programming environment was used for statistical analysis.

**Table 1:** Sociodemographic and clinical characteristics of mother–child dyads (*n* = 2899).

	*n* observations/events (missing)	%/ Mean ± SD	Min, Max
Maternal characteristics			
Maternal age (years)	2899	32.13 ± 5.00	18.00, 49.00
Ethnic identity			
Non-Hispanic	1507	52.0%	–
Hispanic	930	32.1%	–
Missing	462	15.9%	–
Education			
Graduate/professional school	810	28.1%	–
At least some college	980	34.0%	–
No college	643	22.3%	–
Missing	466	15.7%	–
ppBMI (kg/m^2^)	2811 (88)	25.5 ± 5.4	15.3, 56.5
With obesity (≥30 kg/m^2^)	1028 (0)	35.5%	–
GWG (kg)	2779 (120)	14.2 ± 6.8	−21.8, 50.5
EGWG	1128 (131)	40.8%	–
Maternal cigarette smoking	47 (1848)	1.6%	–
Child characteristics			
Infant sex			
Male	1473	50.2%	–
Female	1426	49.2%	–
Gestational age at delivery (weeks)		38.93 ± 1.00	37.00, 41.00
Birth weight (g)	2897 (2)	3431 ± 449.56	1850, 5190
Overweight at 36 months (weight-for-age ≥ 85%ile)	274 (1819)	25.4%	–
With obesity at 36 months (weight-for-age ≥ 95%ile)	113 (1819)	10.5%	–
Growth velocity (Z-score)			
Birth to 6 months	2297 (602)	0.05 ± 1.26	−8.23, 6.91
6–12 months	1983 (916)	0.21 ± 1.04	−7.38, 7.41
12–18 months	1755 (1144)	0.11 ± 0.76	−6.47, 6.19
18–24 months	1481 (1418)	0.04 ± 0.54	−2.33, 3.96
24–36 months	949 (1950)	-0.14 ± 0.57	−2.38, 2.81
Rapid weight gain (Z-score > 0.67)			
Birth to 6 months	690 (602)	30.0%	–
6–12 months	478 (916)	24.1%	–
12 –18 months	256 (1144)	14.6%	–
18–24 months	139 (1418)	9.4%	–
24–36 months	68 (1950)	7.2%	–

*BMI* body mass index, *GWG* gestational weight gain, *EGWG* excess gestational weight gain, *Max* maximum value, *Min* minimum value, *ppBMI* prepregnancy BMI.

## Results

### Sociodemographic and clinical characteristics of study participants

The distributions of maternal and child demographic and clinical factors are provided in Table 1. Mothers were ethnically diverse: *n* = 930 (32.1%) self-reported Hispanic ethnicity and *n* = 1507 (52.0%) reported non-Hispanic, with 15.9% missing ethnicity. Approximately 35.5% of mothers had obesity and 40.8% had EGWG. At 36 months of age, 274 (25.4%) children had overweight and *n* = 113 (10.5%) had obesity. Child growth velocity was greatest between 6 and 12 postnatal months, with 30.0% defined as RWG. Growth velocity was least between 24 and 36 postnatal months, with 7.2% defined as RWG.

### Associations between maternal weight/weight gain and overweight/obesity in children

As shown in Table 2, a 1 kg/m^2^ greater ppBMI (RR = 1.04; 95% confidence interval (CI): 1.03, 1.06) and 1 kg GWG (RR = 1.02; 95% CI: 1.00, 1.04) were associated with a greater risk of childhood OWO at 36 months of age. Maternal obesity status (RR = 1.64; 95% CI: 1.26, 2.13) and EGWG (RR = 1.39; 95% CI: 1.09, 1.74) also predicted a greater risk of childhood OWO. A time trend suggested that the odds of OWO decreased over time, but the associations with ppBMI and GWG remained similar to the main model when also adjusted for year of birth using a logistic regression model (Supplementary Table S1). We also found similar associations when limited to *n* = 861 mother–child dyads without missing covariates (Supplementary Table S2), and when we used inverse probability of selection weights to accommodate loss to follow-up (Supplementary Table S3). We did not include a random effect on mother to account for correlated outcomes among *n* = 299 siblings, which would over-adjust for ppBMI and GWG. However, as shown in Supplementary Table S4, we found similar associations of OWO with ppBMI in *n* = 1012 primiparas, without siblings and *n* = 1887 multiparas, with siblings, suggesting that bias due to correlated outcomes between siblings was modest).

**Table 2:** Risk of overweight/obesity in children at 36 months of age associated with continuous maternal ppBMI and GWG or categorical maternal pre-pregnancy overweight/obesity and EGWG (*n* = 2899).

	RR^a^	95% CI	*P*-value
Continuous predictors			
ppBMI (kg/m^2^)	1.04	1.03, 1.06	<0.0001
GWG (kg)	1.02	1.00, 1.04	0.023
Maternal age (years)	1.02	0.99, 1.04	0.239
Education (ref = college)			
Graduate/professional school	0.93	0.73, 1.66	0.642
No college	1.10	0.69, 1.24	0.599
Hispanic ethnicity (ref = non-Hispanic)	0.82	0.57, 1.19	0.298
Sex (ref = female)	1.19	0.95, 1.49	0.130
Gestational age at delivery (weeks)	1.06	0.94, 1.18	0.343
Categorical predictors			
With obesity (BMI ≥ 30 kg/m^2^)	1.64	1.26, 2.13	0.0002
EGWG	1.39	1.09, 1.74	0.007
Maternal age (years)	1.01	0.99, 1.04	0.264
Education (ref = college)			
Graduate/professional school	0.92	0.69, 1.23	0.575
No college	1.10	0.73, 1.65	0.660
Hispanic ethnicity (ref = non-Hispanic)	0.81	0.56, 1.17	0.255
Sex (ref = female)	1.19	0.95, 1.49	0.131
Gestational age at delivery (weeks)	1.05	0.94, 1.18	0.363

^a^Estimated using multiple log-binomial regression.

*BMI* body mass index, *CI* confidence interval, *EGWG* excess gestational weight gain, *GWG* gestational weight gain, *ppBMI* prepregnancy BMI, *Ref* reference category, *RR* relative risk.

We found that associations for GWG and EGWG varied by maternal ethnicity. Maternal GWG (*P* = 0.023) and EGWG (*P* = 0.079) interacted with ethnicity on OWO such that there was no difference in the odds of OWO associated with a 1 kg GWG or EGWG among mothers who identified as Hispanic, whereas the odds of OWO were 1.04-fold and 1.66-fold greater, respectively, for their non-Hispanic counterparts (Table 3). We further stratified the analysis by both ethnicity (Hispanic and non-Hispanic) and parity (primiparous and multiparous), but results were similar (Supplementary Table S5). There were no significant interactions with infant sex, low birth weight or gestational age at delivery (data not shown).

**Table 3:** Odds of overweight/obesity in children at 36 months of age associated with continuous maternal ppBMI and GWG or categorical prepregnancy obesity and EGWG, adjusted for confounders, by Hispanic ethnicity (*n* = 2899)^a^.

	OR^b^	95% CI	*P*-value
Hispanic women (*n* = 1034)			
Continuous predictors			
ppBMI (kg/m^2^)	1.03	0.99, 1.07	0.196
GWG (kg)	0.99	0.96, 1.03	0.651
Maternal age (years)	1.05	1.00, 1.09	0.039
Education (ref = college)			
Graduate/professional school	0.91	0.32, 2.61	0.859
No college	1.10	0.65, 1.89	0.718
Sex (ref = female)	0.99	0.63, 1.55	0.960
Gestational age at delivery (weeks)	0.90	0.72, 1.12	0.351
Categorical predictors			
With Obesity (BMI ≥ 30 kg/m^2^)	1.54	0.92, 2.59	0.100
EGWG	1.06	0.66, 1.72	0.806
Maternal age (years)	1.04	1.00, 1.09	0.042
Education (ref = college)			
Graduate/professional school	0.94	0.33, 2.61	0.911
No college	1.13	0.66, 1.93	0.650
Sex (ref = female)	0.97	0.62, 1.53	0.909
Gestational age at delivery (weeks)	0.90	0.72, 1.12	0.322
Non-Hispanic women (*n* = 1865)			
Continuous predictors			
ppBMI (kg/m^2^)	1.06	1.035, 1.09	<0.0001
GWG (kg)	1.04	1.015, 1.07	<0.0001
Maternal age (years)	0.99	0.955, 1.03	0.723
Education (ref = college)			
Graduate/professional school	0.95	0.66, 1.35	0.767
No college	1.16	0.46, 2.93	0.744
Sex (ref = female)	1.35	0.99, 1.86	0.062
Gestational age at delivery (weeks)	1.16	0.99, 1.37	0.072
Categorical predictors			
With obesity (BMI ≥ 30 kg/m^2^)	1.88	1.27, 2.79	0.002
EGWG	1.66	1.20, 2.30	0.002
Maternal age (years)	0.99	0.95, 1.03	0.662
Education (ref = college)			
Graduate/professional school	0.94	0.66, 1.34	0.731
No college	1.14	0.46, 2.81	0.775
Sex (ref = female)	1.37	0.99, 1.88	0.054
Gestational age at delivery (weeks)	1.16	0.99, 1.37	0.073

*BMI* body mass index, *CI* confidence interval, *EGWG* excess gestational weight gain, *GWG* gestational weight gain, *OR* odds ratio, *ppBMI* prepregnancy BMI, *ref* reference category.

^a^*P* = 0.023 for interaction between GWG and ethnicity and *P* = 0.079 for interaction between EGWG and ethnicity.

^b^Estimated using multiple logistic regression and averaged across 10 imputed data sets.

### Associations between maternal weight/weight gain and child body weight

**Fig. 1:**
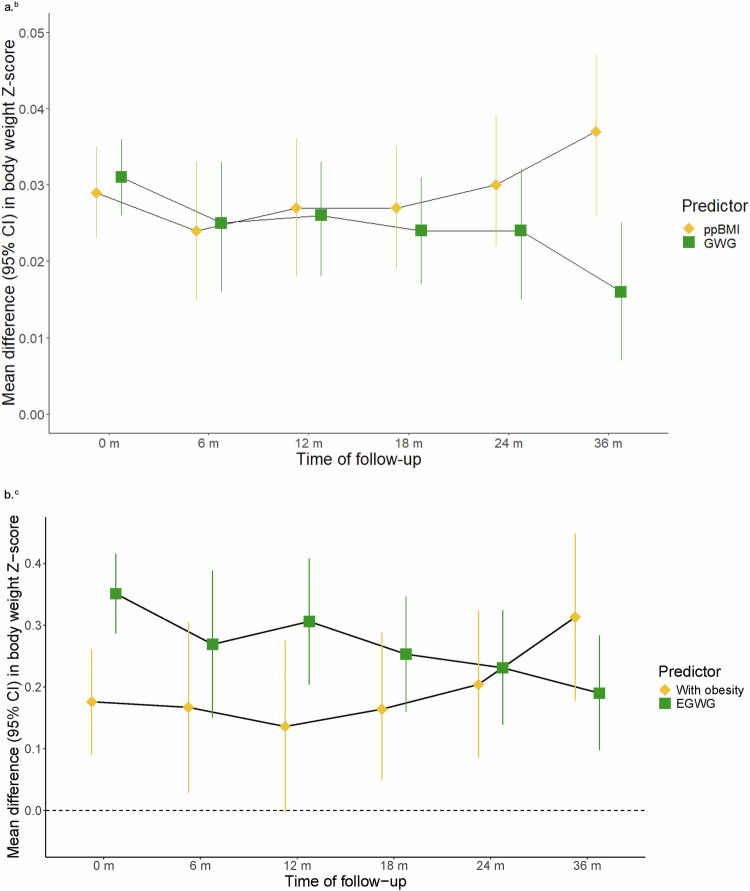
Differences in child weight Z-scores at birth, 6, 12, 18, 24, and 36 months (n = 2899)^a^. **a** Associations at each age with continuous maternal ppBMI and GWG. **b** Associations at each age with categorical maternal prepregnancy obesity and EGWG. All estimates were adjusted for confounders.

As shown in Table 4 ppBMI (mean difference = 0.03, 95% CI: 0.02, 0.04) and GWG (mean difference = 0.02; 95% CI: 0.02, 0.03) independently predicted child body weight Z-score between 0 and 36 months (Table 4). Maternal obesity status (mean difference = 0.19; 95% CI: 0.10, 0.29) and EGWG (mean difference = 0.27; 95% CI: 0.20, 0.34) also predicted greater child body weight Z-score. We detected statistically significant interactions between both ppBMI (*P* = 0.005) and GWG (*P* = 0.001) with time of follow-up such that the associations between ppBMI and body weight Z-score were greater at later times of follow-up than earlier, and conversely, the associations between GWG and body weight Z-score were greater at earlier times of follow-up than later (Fig. 1a). For example, as shown in Supplementary Table S5, the association of body weight Z-score with ppBMI at 36 months) was greater than at birth, whereas the association between body weight Z-score and GWG at 36 months was less than at birth. The trends were similar for maternal obesity status and EGWG as predictors of offspring with OWO over time (Fig. 1b).

**Table 4:** Difference in child weight Z-score through 36 months of age associated with continuous ppBMI and GWG or categorical maternal prepregnancy overweight/obesity and EGWG, adjusted for confounders (*n* = 2899).

	Mean difference^a^	95% CI	*P*-value
Continuous predictors			
ppBMI (kg/m^2^)	0.03	0.02, 0.04	<0.0001
GWG (kg)	0.02	0.02, 0.03	<0.0001
Maternal age (years)	−0.003	−0.01, 0.01	0.465
Education (ref = college)			
Graduate/professional school	−0.002	−0.08, 0.08	0.969
No college	0.17	0.04, 0.289	0.009
Hispanic ethnicity (ref = non-Hispanic)	0.22	0.12, 0.32	<0.0001
Male sex (ref = female)	−0.02	−0.08, 0.05	0.668
Gestational age at delivery (weeks)	0.08	0.05, 0.12	<0.0001
Categorical predictors			
With obesity (BMI ≥ 30 kg/m^2^)	0.19	0.10, 0.29	<0.0001
EGWG	0.27	0.20, 0.34	<0.0001
Maternal age (years)	−0.003	−0.01, 0.01	0.456
Education (ref = college)			
Graduate/professional school	−0.01	−0.09, 0.09	0.884
No college	0.15	0.03, 0.27	0.019
Hispanic ethnicity (ref = non-Hispanic)	0.21	0.11, 0.32	0.0001
Male sex (ref = female)	−0.02	−0.08, 0.05	0.646
Gestational age at delivery (weeks)	0.09	0.06, 0.12	<0.0001

^a^Estimated using multiple linear regression with random intercept on child.

*BMI* body mass index, *CI* confidence interval, *EGWG* excess gestational weight gain, *GWG* gestational weight gain, *ppBMI* prepregnancy BMI.

### Associations between maternal weight/weight gain and child growth velocity

We found an inverse association between GWG and child growth velocity (mean difference = -0.003; 95% CI: −0.01, −0.002), and contradictory associations for ppBMI (RR = 1.01; 95% CI: 1.00, 1.02) and GWG (RR = 0.99; 95% CI: 0.99, 1.00) with RWG, albeit not statistically significant (Supplementary Table S7). Results were similar using maternal obesity status and EGWG as predictors of child growth velocity (Supplementary Table S8). The associations were similar, yet statistically significant for ppBMI and GWG as predictors of RWG, when we limited the analysis to *n* = 1907 mother–child dyads without missing covariates (Supplementary Table S9) and when we used inverse probability of treatment weights to accommodate loss to follow-up (Supplementary Table S10).

### Birthweight and growth velocity as mediators of associations between maternal weight/weight gain and risk of overweight/obesity in children

We employed model-based causal mediation analysis to estimate associations between risk of OWO at 36 months with ppBMI and GWG, using birth weight and growth velocity from 0 to 6 months as intervening variables (Table 5). Approximately 26.8% (95% CI: 14.9%, 55.9%) of the association between ppBMI and OWO was attributed to birth weight as a mediating variable. In contrast, growth velocity from 0 to 6 months did not intervene in the association. We also identified birth weight as a potential mediator, and growth velocity as potential “inconsistent” mediator of the association between maternal GWG and childhood obesity. However, the 95% confidence intervals for the effect estimates were very wide and the total effects were not statistically significant.

**Table 5:** Causal mediation analysis of association between continuous ppBMI or GWG and overweight/obesity in children at 36 months of age, with child birth weight and child growth velocity between 0 and 6 months as intervening variables (*n* = 2899)^a^.

	ppBMI			GWG		
	β^a^	95% CI	*P*-value	β^a^	95% CI	*P*-value
Birth weight (g) is mediating variable						
Total effect	0.001	0.001, 0.002	<0.0001	0.001	−0.001, 0.002	0.28
Indirect effect	0.0004	0.0002, 0.001	<0.0001	0.001	0.0007, 0.002	<0.0001
Direct effect	0.001	0.001, 0.001	0.004	−0.0003	−0.0023, 0.001	0.728
Percent mediated	26.8	14.9, 55.9	<0.0001	133.2	−153.39, 1435.9	0.280
Child growth velocity between 0 and 6 months (Z-score) is mediating variable						
Total effect	0.001	−0.001, 0.002	<0.0001	0.0009	−0.001, 0.002	0.228
Indirect effect	−1.0955 ×10^−5^	−7.37 ×10^−5^, 0.00	0.640	−0.0001	−0.0003, 0.0000	0.036
Direct effect	0.001	0.001, 0.002	<0.0001	0.0010	−0.001, 0.002	0.186
Percent mediated	−0.8	−5.3, 2.7	0.622	−11.33	−136.2, 72.7	0.252

*ppBMI* prepregnancy body mass index, *CI* confidence interval, *GWG* gestational weight gain.

^a^Adjusted for GWG or ppBMI, maternal BMI (kg/m^2^), age (years), education (no college, college = ref, graduate/professional school), ethnicity (Hispanic, non-Hispanic white = ref), child sex (male, female), and gestational age at delivery (weeks) using multiple log binomial regression with random intercept on child. Simulations (*n* = 1000) used to calculate non-parametric bootstrap CIs.

## Discussion

In this prospective study of 2899 children from a 2012–2019 US birth cohort, we found that greater ppBMI and GWG were independently associated with increased risk of early OWO at 36 months of age. While consistent with prior research, our study demonstrates the associations prospectively, in a large, ethnically diverse population. We also found that higher ppBMI and GWG were similarly associated with a higher infant body weight Z-score at delivery, 6 months, and 12 months of age, although with increasing for ppBMI and decreasing magnitude for GWG with infant body weight Z-score between 0 and 36 months of age. We also found that ppBMI and GWG interacted with self-reported maternal ethnicity, such that the associations with OWO at 36 months were stronger among mothers who identified as non-Hispanic compared with those who identified as Hispanic. To our knowledge, this is the first study to identify conditionally independent and divergent patterns of association between maternal ppBMI, GWG and early childhood risk of OWO, as well as the first to document different associations of ppBMI and GWG with childhood OWO among self-reported Hispanic and non-Hispanic mothers.

Our work extends previous studies of prenatal environment and child growth in several ways. In line with prior research, we found that children born to mothers with higher ppBMI or greater GWG experienced an elevated risk of developing obesity [28–32]. However, previous studies had few longitudinal data points to estimate the associations between maternal prenatal adiposity and childhood obesity at specific ages, such as 36 months. Deierlein et al. [33] reported higher child weight z-scores at birth and at 3 years of age among mothers with EGWG compared to those with adequate GWG, a finding similar to ours but based on a much smaller sample (*n* = 476).

Our results suggest that the associations of maternal ppBMI, GWG and EGWG with infant and childhood risk of OWO varied over time. GWG was more strongly associated with outcomes before 6 months, while ppBMI showed stronger associations after 6 months (Fig. 1a). A similar pattern was observed for maternal obesity status and EGWG: EGWG had a stronger association with OWO before 24 months, whereas maternal obesity status was stronger after 24 months (Fig. 1b). This temporal distinction isn’t clearly documented in previous studies, which typically showed static overall associations or focused on broader age windows. One possible explanation is that GWG primarily influences growth in early infancy, whereas maternal ppBMI reflects broader environmental and metabolic factors that exert more persistent effects on child growth throughout early childhood. These findings also suggest that the timing of measurements may influence observed associations and highlight the importance of considering age-specific effects. Future studies with more frequent and standardized longitudinal measurements are needed to clarify these temporal patterns and inform targeted interventions at different stages of early childhood.

Earlier studies found mixed evidence regarding the impact of ppBMI and GWG on growth velocity [32–34]. Deierlein and colleagues reported that EGWG was associated with faster rates of change in both weight and length between 0 and 36 months than adequate GWG [33], whereas Sha and colleagues found associations between ppBMI and GWG and baseline child weight but not the trajectory of weight change for children aged 1–18 months [34]. In this study, we did not identify associations between ppBMI, maternal obesity status, GWG or EGWG and child growth velocity or RWG. Furthermore, child growth velocity did not mediate the associations of ppBMI or GWG with childhood OWO at 36 months.

The associations between maternal adiposity and childhood OWO may vary by maternal ethnicity, and recent work has begun to identify racial–ethnic differences similar to those that we found in our study [13, 35]. While Hunt et al. leveraged a racially and ethnically diverse cohort [13], they did not conduct race/ethnic-specific analyses due to limited power and instead called for future studies to address this gap. Leonard and colleagues did not find a racial or ethnic difference in the link between GWG and childhood OWO at birth, ages 2–5 years, or ages 6–11 years, but did show a difference for teenagers ages 12–19 years [35]. The association between EGWG and childhood OWO was stronger in non-Hispanic white children 12–19 years of age than among non-Hispanic Black and Hispanic children [35]. Similarly, we found null associations between ppBMI, maternal obesity status, GWG and EGWG with childhood OWO among mothers who identified as Hispanic in our study. However, we found that maternal adiposity was associated with higher odds of childhood OWO among non-Hispanic women, and these associations were not explained by parity. In contrast, although the prevalence of OWO was higher among Hispanic than non-Hispanic children, these maternal factors, such as ppBMI and GWG, were not significantly related to their risk of OWO. This discrepancy suggests that for Hispanic children, other or later obesogenic influences—such as postnatal environmental, cultural, or socioeconomic factors—may play a more important role in shaping OWO than prenatal factors.

We found that birthweight was an important mediator of the association between ppBMI and childhood OWO at 36 months, but growth velocity or RWG was not a mediator, although it is a risk factor for children to develop overweight or obesity. Our finding that birth weight acts as a mediator is consistent with previous studies [36–38]. Birth weight partially explained the association between maternal ppBMI and childhood OWO, indicating that there additional pathways are likely. These might include early feeding practices, child diet and activity, maternal metabolic influences and the home environment. Unfortunately, we were unable to test this hypothesis in our study, so a more comprehensive future study is needed for a more definitive interpretation.

Our study has a number of strengths and limitations. Our large and ethnically diverse study population afforded sufficient statistical power to detect modest effects with good precision and to investigate differences among ethnic groups. Our longitudinal study design, including child weight data in six-month intervals from birth to 36 months, ensured temporality between exposure and outcome, precluding a reverse causation bias. Our cohort experienced attrition over time; 33% of enrolled participants responded to the follow-up survey at 36 months. We implemented multiple imputation to retain the enrolled sample size (*n* = 2899) throughout the analysis and found similar results using a complete case without missing data and inverse probability of selection weights in sensitivity analyses, suggesting that our results were robust to loss to follow-up. Child weights were self-reported, which may have misclassified the outcome in some participants. However, a validation substudy (n = 180) found a strong correlation (r = 0.93) between parental report and measured weight at 12 months, so the effect was likely modest [17]. Still, error in self-reported child weights may have introduced bias into the results. Furthermore, we were unable to evaluate children’s BMI-for-age and weight-for-height as study outcomes due to a larger number of missing values for height. A future longitudinal investigation with measurement of children’s weight and height by trained personnel using a standardized protocol will be necessary for a more definitive result. Maternal prepregnancy weight was also self-reported during the second trimester. Prior studies show that self-reported prepregnancy weight is generally reliable, with most women underreporting by only 1–3 kg and BMI categories matching measured values in the majority of cases [39–41]. Nevertheless, some misclassification is possible because maternal weight at the first prenatal visit can vary, and GWG reflects not only maternal adiposity but also fetal weight, amniotic fluid, placenta and fluid shifts related to pregnancy conditions [18, 42]. In addition, the accuracy of self-reported gestational weight gain may differ across ethnic and socioeconomic groups, which could contribute to the ethnic subgroup differences in our results [43]. We also did not have information on infant nutritional choices, such as breastfeeding or early introduction of solid foods, which are important determinants of child OWO. However, these events occurred downstream of (i.e., after) pregnancy, and were unlikely confounders of our results [44]. Still, a future study should incorporate breastfeeding and solid foods as potential mediators and moderators of the associations between maternal obesity and GWG and children’s OWO. Our study population included only singleton term births that did not require NICU admission; therefore, the results may not be generalizable to multiple gestations, preterm births or term births requiring NICU care. Finally, our follow-up concluded at 36 months and so it is unclear if the associations between maternal adiposity and childhood OWO persists at older ages.

## Conclusion

In this large longitudinal birth cohort study, we identified that ppBMI and GWG were independently and positively associated with childhood OWO, and that the relative strengths of the associations were different at earlier and later ages and by maternal self-identified ethnicity. Given the growing prevalence of obesity in childbearing women and the high frequency of excessive weight gain during pregnancy, it is imperative to understand the impact of these prenatal characteristics on subsequent obesity in children to design interventions. Our findings further support the association of the prenatal obesogenic environment association with obesity in children; suggesting that interventional studies to optimize health status before and during pregnancy may help mitigate the risk of overweight and obesity in children.

## Supplementary information

Supplemental Tables

## Data Availability

The data that support the findings of this study are not publicly available due to privacy and ethical restrictions. Data may be available from the corresponding author upon reasonable request and with appropriate institutional approvals.
