# Supplementary material for: The association of prenatal adiposity characteristics with early childhood overweight and obesity: findings from a large and diverse mother–child cohort
Source: Int J Obes (Lond). 2026 Apr 14;50(7):1534–42. doi: 10.1038/s41366-026-02082-7 (PMC13391355; doi:10.1038/s41366-026-02082-7)
Supplement: Supplementary file 1 — Supplemental Tables [file 41366_2026_2082_MOESM1_ESM.docx]

Table S1: Odds of overweight/obesity in children at 36 months of age associated with continuous maternal prepregnancy BMI and gestational weight gain, adjusted for confounders and time trend (n=2899)

| Predictors | OR ^a^ | 95% CI | P-value |
| --- | --- | --- | --- |
| ppBMI (kg/m^2^) | 1.06 | 1.04, 1.08 | <0.001 |
| GWG (kg) | 1.03 | 1.01, 1.05 | 0.021 |
| Maternal age (years) | 1.03 | 0.99, 1.04 | 0.298 |
| Education (ref = college) |  |  |  |
| Graduate/professional school | 0.97 | 0.70, 1.35 | 0.869 |
| No college | 1.01 | 0.64, 1.57 | 0.981 |
| Hispanic ethnicity (ref = non-Hispanic) | 0.87 | 0.58, 1.30 | 0.491 |
| Sex (ref = female) | 1.22 | 0.94, 1.57 | 0.133 |
| Gestational age at delivery (weeks) | 1.06 | 0.93, 1.20 | 0.368 |
| Year of birth | 0.73 | 0.66, 0.80 | <0.0001 |

^a^ Estimated using multiple logistic regression.

BMI, body mass index; CI, confidence interval; GWG, gestational weight gain; OR, odds ratio; ref, reference category; ppBMI, prepregnancy BMI.

Table S2: Odds of overweight/obesity in children at 36 months of age associated with continuous maternal pre-pregnancy BMI and gestational weight gain, adjusted for confounders, among women/children without missing data (n=861) ^a^

| Predictors | OR ^b^ | 95% CI | P-value |
| --- | --- | --- | --- |
| Maternal prepregnancy BMI (kg/m^2^) | 1.07 | 1.04, 1.10 | <0.001 |
| Maternal gestational weight gain (kg) | 1.04 | 1.01, 1.07 | 0.017 |
| Maternal age (years) | 1.02 | 0.98, 1.06 | 0.345 |
| Education (ref = college) |  |  |  |
| Graduate/professional school | 0.83 | 0.57, 1.210 | 0.331 |
| No college | 1.69 | 0.94, 3.06 | 0.081 |
| Hispanic ethnicity (ref = non-Hispanic) | 1.41 | 0.83, 2.37 | 0.195 |
| Male sex (ref = female) | 1.24 | 0.90, 1.71 | 0.184 |
| Gestational age at delivery (weeks) | 0.99 | 0.84, 1.17 | 0.873 |

^a^ This analysis includes n=807 mother-child dyads who returned the 36-month study follow-up questionnaire; ^b^ Estimated using multiple logistic regression.

BMI, body mass index; CI, confidence interval; OR, odds ratio; ref, reference category.

Table S3: Odds of overweight/obesity in children at 36 months of age associated with continuous maternal pre-pregnancy BMI and gestational weight gain, adjusted for confounders, among women/children without missing data, using inverse probability of selection weights (n=861) ^a^

| Predictors | OR ^b^ | 95% CI | P-value |
| --- | --- | --- | --- |
| Maternal prepregnancy BMI (kg/m^2^) | 1.07 | 1.04, 1.10 | <0.001 |
| Maternal gestational weight gain (kg) | 1.03 | 1.00, 1.06 | 0.017 |
| Maternal age (years) | 1.03 | 0.99, 1.07 | 0.345 |
| Education (ref = college) |  |  |  |
| Graduate/professional school | 0.81 | 0.56, 1.18 | 0.331 |
| No college | 1.60 | 0.88, 2.93 | 0.081 |
| Hispanic ethnicity (ref = non-Hispanic) | 1.45 | 0.85, 2.47 | 0.195 |
| Male sex (ref = female) | 1.17 | 0.84, 1.63 | 0.184 |
| Gestational age at delivery (weeks) | 0.94 | 0.79, 1.12 | 0.873 |

^a^ This analysis includes n=807 mother-child dyads who returned the 36-month study follow-up questionnaire; ^b^ Estimated using multiple logistic regression.

BMI, body mass index; CI, confidence interval; OR, odds ratio; ref, reference category.

Table S4: Odds of overweight/obesity in children at 36 months of age associated with continuous ppBMI and GWG, adjusted for confounders, by parity (n=2899)

| Primipara (n=1012) | OR ^a^ | 95% CI | P-value |
| --- | --- | --- | --- |
| ppBMI (kg/m^2^) | 1.07 | 1.03, 1.11 | 0.003 |
| GWG (kg) | 1.05 | 1.02, 1.08 | 0.003 |
| Maternal age (years) | 1.00 | 0.96, 1.04 | 0.863 |
| Education (ref = college) |  |  |  |
| Graduate/professional school | 0.79 | 0.51, 1.24 | 0.294 |
| No college | 1.29 | 0.69, 2.42 | 0.422 |
| Hispanic ethnicity (ref = non-Hispanic) | 1.66 | 0.992, 2.77 | 0.053 |
| Sex (ref = female) | 1.38 | 1.00, 1.90 | 0.122 |
| Gestational age at delivery (weeks) | 1.06 | 0.91, 1.23 | 0.456 |
|  |  |  |  |
| Multipara (n=1887) |  |  |  |
| ppBMI (kg/m^2^) | 1.08 | 1.05, 1.12 | <0.0001 |
| GWG (kg) | 1.03 | 1.00, 1.05 | 0.018 |
| Maternal age (years) | 1.01 | 0.98, 1.05 | 0.018 |
| Education (ref = college) |  |  |  |
| Graduate/professional school | 0.71 | 0.44, 1.13 | 0.136 |
| No college | 1.21 | 0.75, 1.96 | 0.420 |
| Hispanic ethnicity (ref = non-Hispanic) | 1.92 | 1.13, 3.26 | 0.020 |
| Sex (ref = female) | 1.47 | 0.01, 1.12 | 0.006 |
| Gestational age at delivery (weeks) | 0.97 | 0.80, 1.17 | 0.702 |

^a^ Estimated using multiple logistic regression.

BMI, body mass index; CI, confidence interval; GWG, gestational weight gain; OR, odds ratio; ppBMI, prepregnancy BMI; ref, reference category.

Table S5: Odds of overweight/obesity in children at 36 months of age associated with continuous maternal ppBMI and GWG or categorical prepregnancy overweight/obesity status and EGWG, adjusted for confounders, stratified by Hispanic ethnicity and parity (n=2899) ^a^

| **Continuous Predictors** | **OR ^b^** | **95% CI** | **P-value** |
| --- | --- | --- | --- |
| Hispanic women |  |  |  |
| Primiparous |  |  |  |
| ppBMI (kg/m^2^) | 1.01 | 0.92, 1.11 | 0.856 |
| GWG (kg) | 1.04 | 0.97, 1.13 | 0.272 |
| Multiparous |  |  |  |
| ppBMI (kg/m^2^) | 1.03 | 0.93, 1.08 | 0.196 |
| GWG (kg) | 0.98 | 0.94, 1.02 | 0.272 |
| Non-Hispanic women |  |  |  |
| Primiparous |  |  |  |
| ppBMI (kg/m^2^) | 1.04 | 1.00, 1.09 | 0.051 |
| GWG (kg) | 1.05 | 1.01, 1.08 | 0.016 |
| Multiparous |  |  |  |
| ppBMI (kg/m^2^) | 1.08 | 1.04, 1.11 | 3.178x10^-5^ |
| GWG (kg) | 1.03 | 1.00, 1.07 | 0.064 |
|  |  |  |  |
| **Categorical Predictors** | **OR ^b^** | **95% CI** | **P-value** |
| Hispanic women |  |  |  |
| Primiparous |  |  |  |
| With Obesity (> 30 kg/m^2^) | 1.01 | 0.26, 3.91 | 0.991 |
| EGWG | 1.41 | 0.50, 3.99 | 0.517 |
| Multiparous |  |  |  |
| With Obesity (> 30 kg/m^2^) | 1.69 | 0.95, 3.01 | 0.072 |
| EGWG | 1.00 | 0.58, 1.73 | 0.993 |
| Non-Hispanic women |  |  |  |
| Primiparous |  |  |  |
| With Obesity (> 30 kg/m^2^) | 1.46 | 0.78, 2.73 | 0.237 |
| EGWG | 1.55 | 0.96, 2.49 | 0.072 |
| Multiparous |  |  |  |
| With Obesity (> 30 kg/m^2^) | 2.28 | 1.64, 3.82 | 0.002 |
| EGWG | 1.72 | 1.10, 2.69 | 0.017 |

^a^ Adjusted for age, education, newborn sex, and gestational age at delivery; ^b^ Estimated using multiple logistic regression and averaged across 10 imputed data sets.

Table S6: Differences in child weight Z-score at 36, 24, 18, 12, and 6 months of age associated with continuous ppBMI and GWG or categorical maternal pre-pregnancy overweight/obesity status and EGWG, adjusted for confounders (n=2899)

| Follow-up Time Point | Mean difference ^a^ | 95% CI | P-value |
| --- | --- | --- | --- |
| At 36 months |  |  |  |
| ppBMI (kg/m^2^) | 0.04 | 0.03, 0.05 | <0.001 |
| GWG (kg) | 0.02 | 0.01, 0.03 | 0.001 |
|  | 0.31 | 0.18, 0.45 |  |
| With Obesity (BMI ≥ 30 kg/m^2^) | 0.19 | 0.10, 0.28 | <0.001 |
| EGWG |  |  | <0.001 |
|  | 0.03 | 0.02, 0.04 |  |
| At 24 months | 0.02 | 0.02, 0.03 |  |
| ppBMI (kg/m^2^) | 0.20 | 0.09, 0.32 | <0.001 |
| GWG (kg) | 0.23 | 0.14, 0.32 | <0.001 |
|  |  |  |  |
| With Obesity (BMI ≥ 30 kg/m^2^) | 0.03 | 0.02, 0.04 | 0.001 |
| EGWG | 0.02 | 0.02, 0.03 | <0.001 |
|  | 0.16 | 0.05, 0.29 |  |
| At 18 months | 0.25 | 0.16, 0.35 |  |
| ppBMI (kg/m^2^) |  |  | <0.001 |
| GWG (kg) | 0.03 | 0.02, 0.04 | <0.001 |
|  | 0.03 | 0.02, 0.03 |  |
| With Obesity (BMI ≥ 30 kg/m^2^) | 0.14 | -0.00, 0.28 | 0.005 |
| EGWG | 0.31 | 0.20, 0.41 | <0.001 |
|  |  |  |  |
| At 12 months | 0.02 | 0.02, 0.03 |  |
| ppBMI (kg/m^2^) | 0.03 | 0.02, 0.03 | <0.001 |
| GWG (kg) | 0.17 | 0.03, 0.31 | <0.001 |
|  | 0.27 | 0.15, 0.39 |  |
| With Obesity (BMI ≥ 30 kg/m^2^) |  |  | 0.056 |
| EGWG | 0.03 | 0.02, 0.04 | <0.001 |
|  | 0.03 | 0.03, 0.04 |  |
| At 6 months | 0.18 | 0.09, 0.26 |  |
| ppBMI (kg/m^2^) | 0.35 | 0.29, 0.42 | <0.001 |
| GWG (kg) | 0.04 | 0.03, 0.05 | <0.001 |
|  | 0.02 | 0.01, 0.03 |  |
| With Obesity (BMI ≥ 30 kg/m^2^) | 0.31 | 0.18, 0.45 | 0.018 |
| EGWG | 0.19 | 0.10, 0.28 | <0.001 |
|  |  |  |  |
| At 0 months | 0.03 | 0.02, 0.04 |  |
| ppBMI (kg/m^2^) | 0.02 | 0.02, 0.03 | <0.001 |
| GWG (kg) | 0.20 | 0.09, 0.32 | <0.001 |
|  | 0.23 | 0.14, 0.32 |  |
| With Obesity (BMI ≥ 30 kg/m^2^) |  |  | <0.001 |
| EGWG | 0.03 | 0.02, 0.04 | <0.001 |

^a^ Estimated using individual multiple linear regression models for each time point, adjusted for GWG or ppBMI (continuous predictor models), or with obesity or EGWG (categorical predictor models), and maternal BMI (kg/m^2^), age (years), education (no college, vs. college vs. graduate/professional school), ethnicity (Hispanic vs. non-Hispanic white), sex (male vs. female), and gestational age at delivery (weeks) with random intercept on child.

BMI, body mass index; CI, confidence interval; EGWG, excess gestational weight gain; GWG, gestational weight gain; ppBMI, prepregnancy BMI.

Table S7: Difference in child growth velocity through 36 months of age associated with continuous maternal ppBMI and GWG weight gain, adjusted for confounders (n=2899)

| Continuous Z-score as outcome | Mean difference ^a^ | 95% CI | P-value |
| --- | --- | --- | --- |
| ppBMI (kg/m^2^) | 0.001 | -0.002, 0.01 | 0.359 |
| GWG (kg) | -0.003 | -0.01, -0.0002 | 0.038 |
| Maternal age (years) | -0.002 | -0.01, 0.002 | 0.295 |
| Education (ref = college) |  |  |  |
| Graduate/professional school | 0.001 | -0.04, 0.04 | 0.978 |
| No college | 0.03 | -0.02, 0.08 | 0.209 |
| Hispanic ethnicity (ref = non-Hispanic) | 0.06 | 0.01, 0.11 | 0.173 |
| Male sex (ref = female) | 0.03 | -0.003, 0.05 | 0.072 |
| Gestational age at delivery (weeks) | -0.05 | -0.07, -0.04 | <0.001 |
|  |  |  |  |
| Dichotomized Z-score >0.67 as outcome | RR ^b^ | 95% CI | P-value |
| ppBMI (kg/m^2^) | 1.01 | 1.00, 1.01 | 0.053 |
| GWG | 0.99 | 0.99, 1.00 | 0.092 |
| Maternal age (years) | 0.99 | 0.98, 1.00 | 0.040 |
| Education (ref = college) |  |  |  |
| Graduate/professional school | 0.96 | 0.87, 1.06 | 0.388 |
| No college | 1.09 | 0.96, 1.24 | 0.174 |
| Hispanic ethnicity (ref = non-Hispanic) | 1.19 | 1.06, 1.34 | 0.004 |
| Male sex (ref = female) | 1.13 | 1.04, 1.22 | 0.003 |
| Gestational age at delivery (weeks) | 0.92 | 0.88, 0.95 | <0.001 |

^a^ Estimated using multiple linear regression with random intercept on child; ^b^ Estimated using multiple log-binomial regression with random intercept on child.

BMI, body mass index; CI, confidence interval; GWG, gestational weight gain; ppBMI, prepregnancy BMI; ref, reference group;; RR, relative risk.

Table S8: Difference in child growth velocity through 36 months of age associated with categorical maternal obesity and EGWG (n=2899)

| Continuous Z-score as outcome | Mean difference ^a^ | 95% CI | P value |
| --- | --- | --- | --- |
| With Obesity (BMI ≥ 30 kg/m^2^) | 0.03 | -0.02, 0.07 | 0.226 |
| EGWG | -0.03 | -0.07, 0.001 | 0.055 |
| Maternal age (years) | -0.002 | -0.01, 0.002 | 0.336 |
| Education (ref = college) |  |  |  |
| Graduate/professional school | -0.002 | -0.04, 0.04 | 0.911 |
| No college | 0.03 | -0.01, 0.08 | 0.163 |
| Hispanic ethnicity (ref = non-Hispanic) | 0.06 | 0.01, 0.10 | 0.010 |
| Male sex (ref = female) | 0.03 | -0.003, 0.06 | 0.077 |
| Gestational age at delivery (weeks) | -0.05 | -0.07, -0.04 | <0.001 |
|  |  |  |  |
| Dichotomized Z-score >0.67 as outcome | RR ^b^ | 95% CI | P value |
| With Obesity (BMI ≥ 30 kg/m^2^) | 1.09 | 0.98, 1.21 | 0.111 |
| EGWG | 0.96 | 0.88, 1.04 | 0.309 |
| Maternal age (years) | 0.99 | 0.98, 1.00 | 0.055 |
| Education (ref = college) |  |  |  |
| Graduate/professional school | 0.95 | 0.86, 1.05 | 0.300 |
| No college | 1.10 | 0.97, 1.25 | 0.141 |
| Hispanic ethnicity (ref = non-Hispanic) | 1.20 | 1.07, 1.35 | 0.003 |
| Male sex (ref = female) | 1.13 | 1.04, 1.22 | 0.004 |
| Gestational age at delivery (weeks) | 0.92 | 0.88, 0.95 | <0.001 |

^a^ Estimated using multiple linear regression with random intercept on child; ^b^ Estimated using multiple log-binomial regression with random intercept on child.

BMI, body mass index; CI, confidence interval; EGWG, excess gestational weight gain; ref, reference group; RR, relative risk.

Table S9: Difference in child growth velocity through 36 months of age associated with continuous maternal prepregnancy BMI and gestational weight gain, among women/children without missing data (n=1907) ^a^

| Continuous Z-score as outcome | Mean difference ^b^ | 95% CI | P value |
| --- | --- | --- | --- |
| ppBMI (kg/m^2^) | 0.001 | -0.003, 0.01 | 0.603 |
| GWG (kg) | -0.003 | -0.01, 0.0 | 0.100 |
| Maternal age (years) | -0.003 | -0.01, 0.0 | 0.285 |
| Education (ref = college) |  |  |  |
| Graduate/professional school | -0.01 | -0.07, 0.04 | 0.635 |
| No college | 0.09 | 0.0, 0.17 | 0.043 |
| Hispanic ethnicity (ref = non-Hispanic) | 0.07 | -0.002, 0.15 | 0.056 |
| Male sex (ref = female) | 0.03 | -0.01, 0.08 | 0.187 |
| Gestational age at delivery (weeks) | -0.07 | -0.10, -0.05 | <0.001 |
|  |  |  |  |
| Dichotomized Z-score >0.67 as outcome | OR ^b^ | 95% CI | P value |
| ppBMI (kg/m^2^) | 1.01 | 1.00, 1.02 | 0.017 |
| GWG (kg) | 0.99 | 0.98, 1.00 | 0.033 |
| Maternal age (years) | 0.98 | 0.97, 0.99 | 0.009 |
| Education (ref = college) |  |  |  |
| Graduate/professional school | 0.94 | 0.81, 1.09 | 0.453 |
| No college | 1.30 | 1.05, 1.61 | 0.017 |
| Hispanic ethnicity (ref = non-Hispanic) | 1.33 | 1.09, 1.61 | 0.005 |
| Male sex (ref = female) | 1.17 | 1.03, 1.32 | 0.014 |
| Gestational age at delivery (weeks) | 0.88 | 0.83, 0.94 | <0.001 |

^a^ This analysis includes n=1907 mother-child dyads who returned the 6-month, 12-month, 18, month, 24 month, or 36-month study follow-up questionnaire; ^b^ Estimated using multiple linear regression with random intercept on child; ^b^ Estimated using multiple logistic regression with random intercept on child.

BMI, body mass index; CI, confidence interval; GWG, gestational weight gain; OR, odds ratio; ppBMI, prepregnancy BMI; ref, reference group.

Table S10: Difference in child growth velocity through 36 months of age associated with continuous maternal pre-pregnancy BMI and gestational weight gain, among women/children without missing data, using inverse probability of selection weights (n=1907) ^a^

| Continuous Z-score as outcome | Mean difference ^b^ | 95% CI | P value |
| --- | --- | --- | --- |
| ppBMI (kg/m^2^) | 0.002 | -0.001, 0.005 | 0.283 |
| GWG (kg) | -0.004 | -0.01, -0.001 | 0.010 |
| Maternal age (years) | -0.002 | -0.01, 0.002 | 0.314 |
| Education (ref = college) |  |  |  |
| Graduate/professional school | 0.0001 | -0.03, 0.03 | 0.995 |
| No college | 0.06 | 0.01, 0.12 | 0.033 |
| Hispanic ethnicity (ref = non-Hispanic) | 0.03 | -0.01, 0.08 | 0.059 |
| Male sex (ref = female) | 0.02 | -0.01, 0.05 | 0.208 |
| Gestational age at delivery (weeks) | -0.06 | -0.08, -0.05 | <0.001 |
|  |  |  |  |
| Dichotomized Z-score >0.67 as outcome | OR ^b^ | 95% CI | P value |
| ppBMI (kg/m^2^) | 1.02 | 1.00, 1.02 | 0.014 |
| GWG (kg) | 0.99 | 0.98, 1.00 | 0.005 |
| Maternal age (years) | 0.98 | 0.97, 1.00 | 0.028 |
| Education (ref = college) |  |  |  |
| Graduate/professional school | 0.94 | 0.77, 1.08 | 0.394 |
| No college | 1.30 | 1.00, 1.69 | 0.028 |
| Hispanic ethnicity (ref = non-Hispanic) | 01.00 | 0.77, 1.23 | 0.963 |
| Male sex (ref = female) | 1.13 | 0.92, 1.24 | 0.066 |
| Gestational age at delivery (weeks) | 0.89 | 0.79, 0.92 | <0.001 |

^a^ This analysis includes n=1907 mother-child dyads who returned the 6-month, 12-month, 18, month, 24 month, or 36-month study follow-up questionnaire; ^b^ Estimated using multiple linear regression with random intercept on child; ^b^ Estimated using multiple logistic regression with random intercept on child.

BMI, body mass index; CI, confidence interval; GWG, gestational weight gain; OR, odds ratio; ppBMI, prepregnancy BMI; ref, reference group.
